# A domain knowledge-based interpretable deep learning system for improving clinical breast ultrasound diagnosis

**DOI:** 10.1038/s43856-024-00518-7

**Published:** 2024-05-17

**Authors:** Lin Yan, Zhiying Liang, Hao Zhang, Gaosong Zhang, Weiwei Zheng, Chunguang Han, Dongsheng Yu, Hanqi Zhang, Xinxin Xie, Chang Liu, Wenxin Zhang, Hui Zheng, Jing Pei, Dinggang Shen, Xuejun Qian

**Affiliations:** 1 School of Mathematics, Xi’an University of Finance and Economics, Xi’an, China; 2 School of Biomedical Engineering, ShanghaiTech University, Shanghai, China; 3 Department of Neurosurgery, Beijing Friendship Hospital, Capital Medical University, Beijing, China; 4 Department of Ultrasound, The First Affiliated Hospital of Anhui Medical University, Hefei, China; 5 Department of Ultrasound, Xuancheng People’s Hospital, Xuancheng, China; 6 Department of General Surgery, The First Affiliated Hospital of Anhui Medical University, Hefei, China; 7 Department of Ultrasound, Peking University Third Hospital, Beijing, China; 8 Department of Breast Surgery, The First Affiliated Hospital of Anhui Medical University, Hefei, China; 9 State Key Laboratory of Advanced Medical Materials and Devices, ShanghaiTech University, Shanghai, China; 10 Shanghai United Imaging Intelligence Co., Ltd., Shanghai, China; 11 Shanghai Clinical Research and Trial Center, Shanghai, China

**Keywords:** Breast cancer, Diagnosis

## Abstract

**Background:**

Though deep learning has consistently demonstrated advantages in the automatic interpretation of breast ultrasound images, its black-box nature hinders potential interactions with radiologists, posing obstacles for clinical deployment.

**Methods:**

We proposed a domain knowledge-based interpretable deep learning system for improving breast cancer risk prediction via paired multimodal ultrasound images. The deep learning system was developed on 4320 multimodal breast ultrasound images of 1440 biopsy-confirmed lesions from 1348 prospectively enrolled patients across two hospitals between August 2019 and December 2022. The lesions were allocated to 70% training cohort, 10% validation cohort, and 20% test cohort based on case recruitment date.

**Results:**

Here, we show that the interpretable deep learning system can predict breast cancer risk as accurately as experienced radiologists, with an area under the receiver operating characteristic curve of 0.902 (95% confidence interval = 0.882 – 0.921), sensitivity of 75.2%, and specificity of 91.8% on the test cohort. With the aid of the deep learning system, particularly its inherent explainable features, junior radiologists tend to achieve better clinical outcomes, while senior radiologists experience increased confidence levels. Multimodal ultrasound images augmented with domain knowledge-based reasoning cues enable an effective human-machine collaboration at a high level of prediction performance.

**Conclusions:**

Such a clinically applicable deep learning system may be incorporated into future breast cancer screening and support assisted or second-read workflows.

## Plain Language Summary

Breast cancer is one of the most common cancers, and finding it early can greatly improve patients’ chances of survival and recovery. We create a tool based on artificial intelligence (AI)—whereby computer software learns to perform tasks that normally require human thinking—called MUP-Net. MUP-Net can analyze medical images to predict a patient’s risk of having breast cancer. To make this AI tool usable in clinical practice, we enabled doctors to see the reasoning behind the AI’s predictions by visualizing the key image features it analyzed. We showed that our AI tool not only makes doctors more confident in their diagnosis but also helps them make better decisions, especially for less experienced doctors. With further testing, our AI tool may help clinicians to diagnose breast cancer more accurately and quickly, potentially improving patient outcomes.

## Abstract (web summary)

Yan, Liang, Zhang et al. propose a domain knowledge-based interpretable deep learning system to improve breast cancer risk prediction from multimodal ultrasound images. Its inherent interpretability enables effective human-machine collaboration and thus may aid clinical decision-making.

## Introduction

Breast cancer is a leading cause of cancer mortality among women and an ongoing threat to global health. It is estimated that in 2022, 287,850 new cases of breast cancer in females would be diagnosed in the United States, continuing with a slow but steady increase of approximately 0.5% in incidence rates per year^1^. Early detection of breast cancer can potentially improve patient outcomes, prompting widespread clinical recommendation for screening mammography to reduce its morbidity^2^. However, mammography exhibits low sensitivity in dense breast tissue, and is not universally accessible across all countries^3^. Ultrasound (US), a low-cost, non-invasive, no-ionizing-radiation, and widely-available imaging modality, serves as a supplementary modality to mammography in screening settings^4,5^ and as the primary imaging modality for characterizing breast masses (i.e., solid or cystic)^6,7^. While the American College of Radiology has established the Breast Imaging Reporting and Data System (BI-RADS) guideline to standardize breast imaging terminology and report management, the intra- and inter-variations among observers in breast US image interpretations still exist^8,9^. In addition, high false positive rates and false negative rates in breast US examinations have limited its applicability to a broader range of screening and diagnostic population.

Artificial intelligence (AI) has been leveraged for many years to alleviate these challenges to some extent^10,11^. With the rapid development of deep learning^12–15^, the convolutional neural network has gradually become a promising approach in the field of breast US image analysis, including region detection^16,17^, lesion segmentation^18,19^, tumor classification^20^ as well as multi-stage tasks^21,22^. Early works primarily focused on the US B-mode image without recognizing the importance of jointly utilizing comprehensive semantic information from other types of US images. A significantly better breast cancer risk prediction has been recently demonstrated via either the combination of US B-mode and colour Doppler^23,24^, or the fusion of US B-mode and elastography^25^. More advance, we demonstrated a clinically applicable deep learning system^26^ could prospectively assess clinical relevant multimodal US images (i.e., B-mode, colour Doppler, and elastography) with non-inferior sensitivity and specificity to experienced radiologists.

Despite the radiologist-level classification of breast cancer, the remaining pivotal issue that needs to be addressed before its clinical deployment is the black-box nature of deep learning^27,28^. Current deep learning systems provide clinicians with the malignant probability results, which the clinicians can either simply trust in plausibility or override. In other words, the potential inferential logic of the deep learning, particularly as a clinical decision-supporting system, is not fully understood for radiologists. Post hoc-based interpretable strategies such as saliency maps^29^, deconvolution^30^, and activation maximization^31^ have been extensively explored to visualize the inner working mechanism of deep learning. However, these approaches still cannot explain how a model exploits these cues.

In this study, we propose a novel interpretable AI system, namely the Multimodal Ultrasound Prototype Network (MUP-Net), using domain knowledge-based prototypes to predict the malignancy risk probability. We demonstrate that MUP-Net has comparable breast cancer prediction performance to the state-of-the-art black-box deep learning models, reaching the level of experienced radiologists in our prospective reader study. The most important contribution of our AI system is the emphasis of human-machine collaboration through its inherent interpretability from pathology-confirmed images, instead of unseen images with post-hoc explainability. In our AI-assisted reader study, we demonstrate the potential of our MUP-Net in aiding clinicians, such as increasing the confidence levels of radiologists and diminishing the discrepancy.

## Methods

### Ethical approval

This study was approved by the Institutional Review Board of the First Affiliated Hospital of Anhui Medical University and Xuancheng People’s Hospital of China. Participants were informed of all aspects of the study, even if it involved only minimal risk. The consents were informed and written in advance. The de-identification procedure was performed before transferring to our study.

### Ultrasound dataset

We prospectively collected US images, including B-mode, colour Doppler, and elastography images, from women with breast lesions in either The First Affiliated Hospital of Anhui Medical University or Xuancheng People’s Hospital of China from August 2019 to December 2022. Detailed collection procedures, inclusion and exclusion criteria for patient recruitment are depicted in Supplementary Fig. 1. The US examinations were performed by one of six breast radiologists (i.e, breast radiologists do all ultrasound scans, instead of breast ultrasound technologists), each with more than 10 years of experience in breast US, using the Aixplorer US scanner (SuperSonic Imagine) with an SL15-4 MHz or SL10-2 MHz linear array transducer following the standard protocol. For each breast, paired US images at the largest long-axis cross-section plane of the lesion were saved. As a result, 4,320 paired US images from 1,440 lesions (464 positives for cancer) from 1,348 patients were collected. The manual review of the pathology note served as the ground-truth labels. Table 1 shows patient demographics and breast lesion characteristics of our dataset. The dataset was split into an 8:2 ratio based on case recruitment date: development cohort (70% for training and 10% for validation), and test cohort (20% for testing).

**Table 1:** Patient demographics and breast lesion characteristics

	Development cohort	Test cohort	Clinical test set
**Number of patients**	1087	261	120
**Age (mean)**	44.6 (18–85)	44.7 (20–80)	44.3 (20–78)
**Number of lesions**	1152	288	120
**BI-RADS category**^**a**^			
3 or lower	80 (6.9%)	24 (8.3%)	9 (7.5%)
4a	648 (56.3%)	132 (45.9%)	48 (40.0%)
4b	236 (20.5%)	56 (19.5%)	27 (22.5%)
4c	109 (9.5%)	39 (13.5%)	19 (15.8%)
5	79 (6.8%)	37 (12.8%)	17 (14.2%)
**Lesion size (mm)**			
<10	351 (30.5%)	65 (22.6%)	27 (22.5%)
10–20	504 (43.7%)	124 (43.1%)	48 (40.0%)
> 20	297 (25.8%)	99 (34.3%)	45 (37.5%)
**Location**			
Upper outer quadrant	549 (47.7%)	139 (48.3%)	53 (44.1%)
Lower outer quadrant	232 (20.1%)	64 (22.2%)	29 (24.2%)
Upper inner quadrant	220 (19.1%)	58 (20.1%)	29 (24.2%)
Lower inner quadrant	94 (8.2%)	18 (6.3%)	6 (5.0%)
Central	57 (4.9%)	9 (3.1%)	3 (2.5%)
**Pathology notes**			
Invasive carcinoma	199 (17.3%)	77 (26.7%)	31 (25.8%)
Carcinoma in situ	38 (3.3%)	16 (5.6%)	9 (7.5%)
Other malignant^b^	98 (8.5%)	36 (12.5%)	20 (16.7%)
Fibroadenoma	349 (30.3%)	65 (22.6%)	25 (20.8%)
Other benign^c^	468 (40.6%)	94 (32.6%)	35 (29.2%)

^a^The BI-RADS category is determined by breast US images only. Pathology results are available for the patients classified as BI-RADS 3 or lower following breast US due to either classification as BI-RADS 4a or higher following mammography or magnetic resonance imaging or requests from patients themselves. ^b^Includes specific malignant results. ^c^Includes adenosis, hyperplasia, mastitis, benign phyllodes tumors, and papillomas.

The breast US images were pre-processed^26^ by a custom annotation tool to remove irrelevant information, such as text and instrument settings. In clinical practice, radiologists are required to manually place a sampling box to select the vascularity (via US colour Doppler image), elasticity (via US elastography image) measurements, as well as corresponding box region for the US B-mode image during the US image acquisition. Guided by these box regions, experienced radiologists adjusted the segmentation masks to ensure the similar lesion-to-mask ratios in each imaging mode, followed by cropping operations.

Owing to the chronological data partition and patient population distribution in the real world, the training cohort has an imbalanced data distribution with 335 malignant and 817 benign lesions. To mitigate this issue, we implemented data augmentation techniques, including horizontal flipping, random rotation, and Gaussian blurring to increase the size of malignant samples. We additionally augmented data on-the-fly during the training by contrast adjustment, horizontal flipping, and random rotation. For the reader study, we randomly and equally selected 120 out of 288 lesions, resulting in 60 benign and 60 malignant cases.

### Interpretable deep learning model

**Fig. 1:**
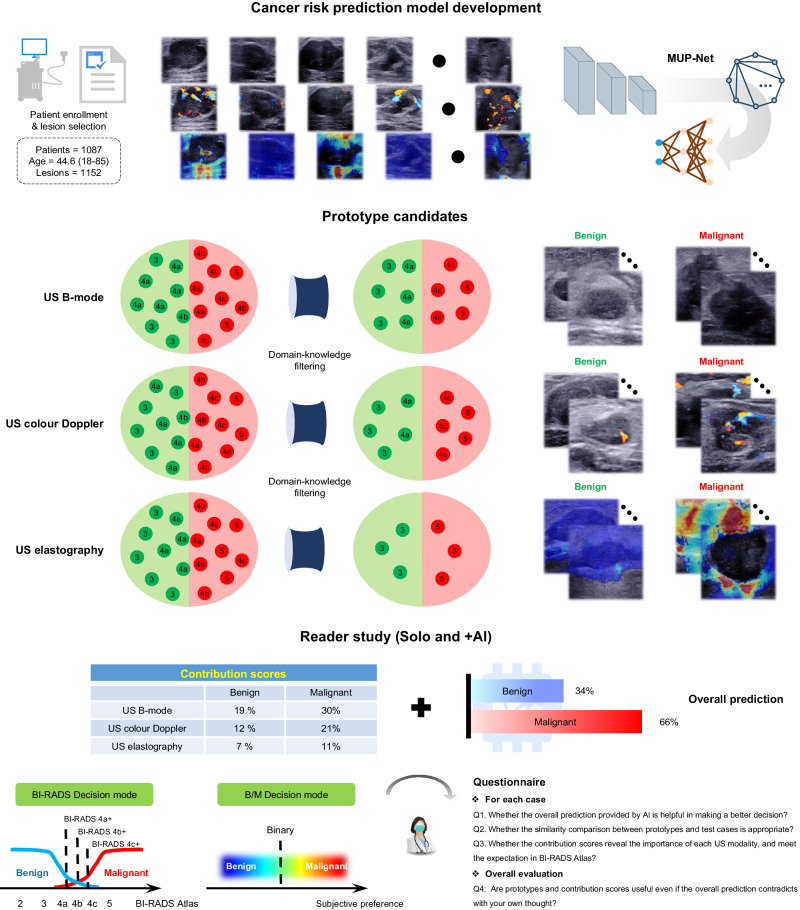
Overall study design of the interpretable AI system. Prospectively collected multimodal ultrasound (US) images, including B-mode, colour Doppler, and elastography were used to develop our MUP-Net model, by utilizing domain knowledge to supervise the selection of prototype candidates. The overall malignancy risk probability and six individual contribution scores generated by the AI system were provided to radiologists as clinical decision-supporting parameters. Two decision modes (i.e., BI-RADS rating and B/M (Benign/Malignant) preference) and a questionnaire were proposed to assess the advantageous use of explainable features by radiologists in making clinical decisions.

The design of our interpretable AI system and the details of MUP-Net are depicted in Fig. 1 and Supplementary Fig. 2, respectively. The MUP-Net is trained using multimodal US images and biopsy-confirmed pathology labels. Three independent backbone networks, namely ResNet-18 (pre-trained on ImageNet), are used to distill semantic features from different US modalities, which are consistent with our previous work^26^. Each patch of the generated feature map is compared against learned prototypes to identify the most similar matches, followed by a quantitative presentation of the similarity scores. These scores are then fed into the final fully connected layer with softmax output to predict a malignancy risk probability. To present explainable features to readers, these similarity scores are converted into contribution scores by combining the associated weights from the last fully connected layer.

Clinical domain knowledge is used for supervising MUP-Net in learning prototypes, which are representative benign and malignant cases for each modality selected from the training data during a MUP-Net optimization process. To diminish the bias introduced by automatic prototype selection, we implemented US domain knowledge to constrain the prototype selection to a subset BI-RADS group. Specifically, we first excluded BI-RADS 4b from the candidate prototypes because it represents a moderate suspicion of malignancy with borderline probability according to the latest BI-RADS Atlas and physician observations in clinical practice. As a result, the biopsy-confirmed benign and malignant prototypes for B-mode and colour Doppler modalities were selected from BI-RADS 3,4a *versus* 4c,5 cases, respectively. Next, the World Federation for Ultrasound in Medicine & Biology guidelines^32^ established a stricter operation for US elastography (i.e., lightly touching the skin and trying not to apply pressure), which implies that the distorted elastography images with atypical appearance are inevitable in clinical practice. Therefore, biopsy-confirmed benign and malignant prototypes for US elastography were exclusively selected from BI-RADS 3 versus 5.

The implementation of the deep learning model is described as follows:

Let $D=\left[X,Y\right]$ denotes the dataset to train the deep learning model. $X$ is a multimodal US image set $\{ {{x}_{i}}^{m} \}_{{i=1},{m=1}}^{D,M}$ where $i$ is a data index, ranging from 1 to $D$. $m$ is a modal index, ranging from 1 to $M$. The modality count $M$ equals to 3 in this work. $Y$ is a binary label set ${\{{y}_{i}\}}_{i=1}^{D}$ indicating the type of lesions. As illustrated in Supplementary Fig. 2, MUP-Net first applies three independent CNN ${f}^{m}\left(\cdot \right)$ on input images ${x}_{i}^{m}$ from different modalities to extract features:

1 $${z}_{i}^{m}={f}^{m}\left({x}_{i}^{m}{{{{{\rm{;}}}}}}{w}^{m}\right)$$

Let $W={\left\{{w}^{m}\right\}}_{m=1}^{M}$ denotes the trainable parameter sets from all ${f}^{m}$. The resulting feature maps ${z}_{i}^{m}\in {R}^{H\times W\times C}$ serve as the input of the prototypical part. Prototypical part aims to learn some meaningful representations of each class in the latent space. Here, MUP-Net learns a set ${P}^{m}$ containing a pre-determined number of prototypes (i.e., $N$) for the $m$-th modality, i.e., ${P}^{m}={\{{p}_{j}^{m}\}}_{j=1}^{N}$ where ${p}_{j}^{m}$ is the $j$-th prototype. To be specific, prototypes ${p}_{j}^{m}$ are the trainable variables of shape ${H}^{m}\times {W}^{m}\times C$. Thus, MUP-Net generates a set $P$ containing $M* N$ prototypes in total, i.e., $P={\left\{{P}^{m}\right\}}_{m=1}^{M}={\{{p}_{j}^{m}\}}_{m=1,j=1}^{M,N}$. For each class $k\in K$, it is represented by ${N}_{k}^{m}$ prototypes in the $m$-th modality, which means $N={\sum }_{k=1}^{K}{N}_{k}^{m}$. We let ${P}^{m,k}\subseteq {P}^{m}$ denote the subset of prototypes allocated to class $k$ in modality $m$.

Since the down sampling operations are performed in backbone ${f}^{m}$, the spatial dimension of a convolution output ${z}_{i}^{m}$ is small. A relatively large receptive field is presented in each pixel of ${z}_{i}^{m}$. Thus, we speculate that a single pixel prototype could be sufficient to represent a significant feature of the original image when mapped back to the original pixel space, i.e., ${H}^{m}={W}^{m}=1$. Therefore, with ${H}^{m} < H,{W}^{m} < W$ and a shared channel number $C$, our model can evaluate the similarity between each prototype ${p}_{j}^{m}$ to/with all pixels of the distilled feature map ${z}_{i}^{m}$, i.e., ${pixels}({z}_{i}^{m})$, in a non-overlapping sliding window manner. In particular, MUP-Net uses Euclidean distances and top-k average pooling to calculate the similarity score for each prototype:

2 $${s}_{{ij}}^{m}={avg}({{top}}_{k}(\log \left(\frac{{d}_{{ij}}^{m}+1}{{d}_{{ij}}^{m}+\varepsilon }\right))),$$

where ${d}_{{ij}}^{m}={|{p}_{j}^{m}-{pixels}({z}_{i}^{m})|}_{2}^{2}$. A small number $\varepsilon$ is used to avoid dividing by zero error. At last, the similarity score set $S=\{ {{s}_{{ij}}}^{m} \}_{{m=1},{j=1}}^{M,N}$ is flattened and fed into the last fully-connected layer $h\left(\cdot \right)$ to produce output logits:

3 $${l}_{i}=h\left({S;}{w}_{h}\right),$$

where ${w}_{h}\in {R}^{{MN}\times K}$ denotes a trainable weight matrix. Indeed, the elements in ${w}_{h}$ indicate the connection weight between a similarity score and its contribution to the output logit of a specific class. These weights are not randomly initialized. Specifically, given a class $k$ and a modality $m$, we set ${w}_{h}^{\left(m* N+j,k\right)}=1$ for the $j$-th prototype ${p}_{j}^{m}$ belonging to ${P}^{m,k}$, and ${w}_{h}^{\left(m* N+j,k\right)}=-0.5$ otherwise. By following such an initialization approach, we can guide MUP-Net to assign a fixed number of prototypes for each predicted class. When ${w}_{h}^{\left(m* N+j,k\right)}$ is positive and the input image ${x}_{i}^{m}$ belongs to class $k$, the fully-connected layer $h$ will let the class $k$ prototypes make a positive contribution to the class $k$ output logit. On the contrary, a negative ${w}_{h}^{\left(m* N+j,k\right)}$ will let the non-class $k$ prototypes make an inverse contribution to the logit of ${y}_{i}$.

Three types of loss functions are combined as the learning target of MUP-Net. The standard cross-entropy is prevalent to supervise classification tasks. It is denoted by ${L}_{{ce}}={CrossEntropy}\left({l}_{i},{y}_{i}\right)$ in this work. The clustering loss and separation loss are applied to guide the learning process of prototypical representation in latent space. For the $m$-th modality, clustering loss is used to encourage some pixels of ${z}_{i}^{m}$ being close to the prototypes contained in ${P}^{m,{y}_{i}}$ which could be defined as:

4 $${L}_{{clu}}={\sum }_{m=1}^{M}{\sum }_{i=1}^{n}{\min }_{{p}_{j}^{m}\in {P}^{m,{y}_{i}}{{{\rm{;}}}}z\in {patch}\left({{{{\boldsymbol{z}}}}}_{i}^{m}\right)}\frac{{\left|z-{p}_{j}^{m}\right|}^{2}}{n}$$

On the contrary, separation loss encourages each patch of ${z}_{i}^{m}$ to stay away from prototypes belonging to ${P}^{m,K\setminus \{{y}_{i}\}}$. It is defined as:

5 $${L}_{{sep}}=-{\sum }_{m=1}^{M}{\sum }_{i=1}^{n}{\min }_{{p}_{j}^{m}\in {P}^{m,K\setminus \{{y}_{i}\}}{{{\rm{;}}}}z\in {patch}\left({{{{\boldsymbol{z}}}}}_{i}^{m}\right)}\frac{{\left|z-{p}_{j}^{m}\right|}^{2}}{n}$$

In addition, L1-norm is applied on ${w}_{h}$ to generate regularization loss ${L}_{{reg}}$. In summary, the overall learning target of MUP-Net is the sum of the above loss functions:

6 $$L={L}_{{ce}}+\alpha {L}_{{sep}}+\beta {L}_{{clu}}+\gamma {L}_{{reg}}$$

where $\alpha$, $\beta ,$ and $\gamma$ were empirically set to 0.8, −0.08, and 1e-4, respectively.

The optimizing procedure of MUP-Net follows the similar steps in the previous works^33,34^. The prototypes in $P$ were randomly initialized using the uniform distribution before training. In the first stage, three independent feature extractors ${f}^{m}\left(\cdot \right)$ and the prototype set $P$ were optimized by network back-propagation. In the second stage, each ${P}^{m,k}$ was projected onto (or replaced by) the nearest latent training patch from the training set belonging to class $k$. The projection not only affects the classification accuracy, but also allows the visualization of the prototypes as training image patches. In the third stage, only the matrix ${w}_{h}$ of the last fully-connected layer $h\left(\cdot \right)$ was trained to adjust the connection weight between an output logit and the similarity scores $S$. These three stages were repeated throughout the whole training process.

### Model development and evaluation

Transfer learning was first applied to the feature extraction part via a ResNet-18 network pre-trained on ImageNet. Adam optimizer was chosen to optimize MUP-Net. We initially set the number of training epochs to 80 and the learning rate to 0.001. The learning rate decreased by 90% every 5 epochs. The batch size was 40. ReLU was chosen as the activation function. The weight decay of factor 0.001 was applied to the parameters of batch normalization layers. Our model was trained on NVIDIA GTX A100 GPU using Python and PyTorch toolbox. During training, data augmentation techniques were applied to speed up convergence and avoid overfitting. In particular, the input images were flipped horizontally with a 50% probability. Brightness and contrast were randomly varied by a factor between [0.9, 1.1]. A random portion of an image between 0.9 and 1 was cropped and resized to its original size. All images were randomly rotated by a degree between −10° and 10°.

Some off-the-shelf deep learning models^35–37^ (i.e., VGG, ResNet, and DenseNet) were trained to compare the performance between black-box models and our interpretable MUP-Net. Their overall architectures were similar, with independent backbone networks used to extract features from different US modalities, and fully connected layers with softmax output used to predict malignancy risk probability. To ensure a fair comparison, the same pre-trained weights from ImageNet, identical data augmentation techniques, and an initial learning rate of 0.001 were applied.

### Reader study and AI-assisted reader study

Nine radiologists (R1-R9) participated in our reader study. We formed two subgroups to investigate the benefits of the AI interpretability in aiding human experts: a senior radiologist group of readers (R1, R5, and R7 with an average of 14 years of clinical experience) and a junior radiologist group of readers (R2, R8, and R9 with a maximum of 2 years of clinical experience).

A two-phase study was conducted to compare the performance between the AI and clinicians. In phase I (reader study), readers were blinded to the original radiologist’s interpretation (i.e., the radiologist who collected and stored the US images) and MUP-Net prediction. Readers independently reviewed test samples and determined BI-RADS ratings along with a Benign/Malignant (B/M) preference. In phase II (AI-assisted reader study), additional explainable features (i.e., matched prototypes and contribution scores) and malignancy risk probability generated by the deep learning model were provided to the readers. Each reader has the opportunity to alter their original BI-RADS ratings or B/M preference from Phase I. In addition to these decision-making tasks, readers were requested to complete a questionnaire expressing their subjective attitudes towards AI assistance.

The questionnaire includes three individual questions (Q1, Q2, and Q3) and an overall evaluation (Q4) for each test case as shown in Fig. 1. Q1 is related to the overall breast cancer risk prediction provided by the final output of the deep learning system. The likelihood of malignancy provides an obvious AI decision without requiring further analysis by radiologists. Q2 and Q3 are attributed to the explainability of our prototypes. Specifically, Q2 checks whether there is a correlation between biopsy-confirmed prototypes and test samples for each modality. Such a similarity comparison is in line with the daily workflow of radiologists (i.e., interpretation based on experience from previously seen US images). Q3 is an extension of Q2 by requesting if the contribution scores reveal the importance of each modality. For instance, as described in BI-RADS Atlas, US B-mode is the dominant modality, while US colour Doppler and US elastography partially provide assorted feature information. In other words, we expect the contribution score of the prototypes to be consistent with this prior. Q4 is an overall assessment to conclude the effectiveness of the AI in increasing clinician confidence levels or making a better clinical decision, or both.

### Statistical analysis

The area under the curve (AUC) of the receiver operating characteristic curve (ROC) value was used to express the performance of the model. The 95% confidence intervals (CI) were calculated based on a non-parametric procedure with 1,000 bootstraps. Delong’s test was used to compare the performance among different AI models. McNamar’s test was used to compare readers’ decisions with and without AI support. *P* < 0.05 was considered to have a statistically significant difference. All statistical analyses were performed using Python (version 3.9) and the statsmodels library (version 0.14.0).

### Reporting summary

Further information on research design is available in the Nature Portfolio Reporting Summary linked to this article.

## Results

Our interpretable AI system was developed from a prospectively enrolled breast US dataset consisting of 4320 US images (paired B-mode, colour Doppler, and elastography) of 1,440 lesions (464 biopsy-confirmed cancer positive) from 1348 patients. All lesions were allocated to 70% training cohort, 10% validation cohort, and 20% test cohort based on case recruitment date. Our proposed AI system not only generates an overall malignancy probability for automated breast cancer risk prediction but also creates inherent explainable features such as representative prototypes and contribution scores, all of which enable a human-understandable interaction as a clinical decision-supporting system.

### Selections of prototypes and performance of the MUP-Net model

The domain knowledge of breast US is incorporated into MUP-Net to refine the selection criteria of prototype candidates. To be specific, regarding the US B-mode and colour Doppler images, benign and malignant prototypes were picked out from biopsy-confirmed cases with BI-RADS rating of 3 & 4a *versus* 4c & 5, respectively. The borderline BI-RADS 4b cases were excluded due to its uncertainty. In terms of US elastography, a stricter preference was applied to the selection process of prototype candidates (i.e., BI-RADS 3 for benign, and BI-RADS 5 for malignant) for the purpose of avoiding potential imaging artifacts caused by human operators^32^. Under this setting, MUP-Net was trained to learn more discriminative cases as benign and malignant prototypes, thus generating reasonable explainable features matched to domain knowledge used by radiologists in clinical settings.

We evaluated the effectiveness of the MUP-Net in three ways. First, we assessed the model’s performance with various prototype numbers, ranging from 6 to 14. As a consequence, our model achieved the best performance on the validation cohort when 10 prototypes were learned for each modality (Supplementary Table 1). Second, we compared MUP-Net with three prevalent black-box models (i.e., VGG-16, ResNet-18 and ResNet-152, DenseNet-121 and DenseNet-201) as shown in Supplementary Fig. 3. Although a few metrics of ResNet and DenseNet families were slightly higher, there was no significant difference between MUP-Net and black box models (*P* < 0.05) on the validation cohort. Third, our MUP-Net achieved the AUC of 0.902 (95% CI = 0.882–0.921), sensitivity of 75.2%, specificity of 91.8%, and F1 score of 0.812 on the test cohort, which was comparable to that of validation cohort (Supplementary Tables 1-3).

In terms of the trained MUP-Net (10 prototypes per modality), we additionally evaluated the effectiveness of three US modalities on the validation cohort by randomly altering one of the US inputs by certain ratios. The changes of performance in Supplementary Table 4 and Supplementary Fig. 4 indicate that the B-mode is essential to improve the sensitivity while elastography mainly improves the specificity of MUP-Net. In other words, B-mode is helpful to identify malignant lesions while the elastography inhibits the occurrence of false positives. The role of colour Doppler is intermediate between B-mode and elastography.

### Reader study

To further investigate the performance of our AI system, we conducted a reader study with nine radiologists who had varying years of experience (1 to 18 years, average of 6 years). In particular, radiologists with more than 10 years of experience are formed to a senior group, while junior group includes radiologists with no more than 2 years of experience. For each test case in the reader study, the readers were asked to independently provide a routine BI-RADS rating and a forced B/M preference using the paired multimodal breast US images. To convert the BI-RADS rating to readers’ sensitivity and specificity, we generated three BI-RADS scores: BI-RADS 4a + , 4b + , and 4c + , which were implemented to match the conditions of BI-RADS 3 *versus* 4a + , BI-RADS 3,4a *versus* 4b + , and BI-RADS 3,4a,4b *versus* 4c + , respectively.

**Fig. 2:**
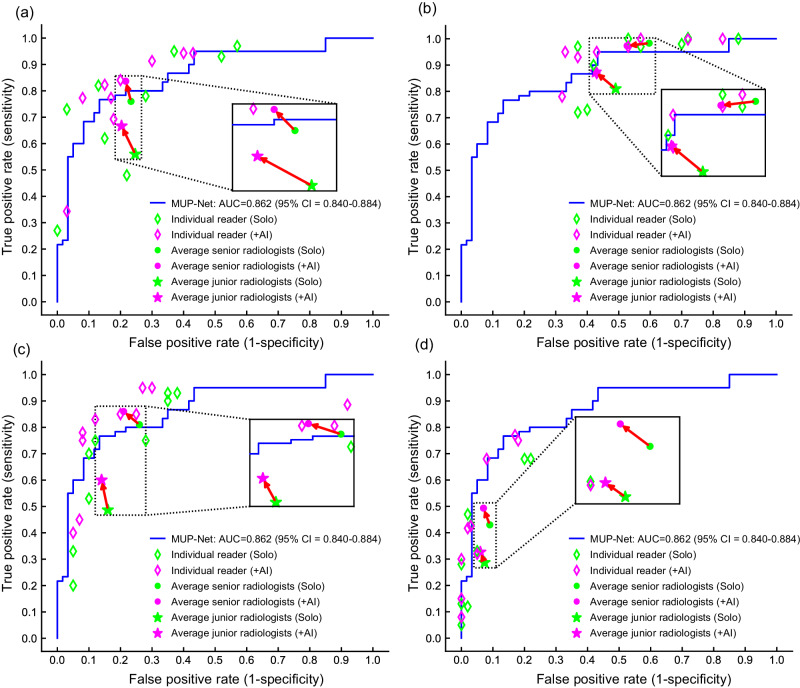
Performance comparison between MUP-Net and readers in predicting breast cancer risk on the clinical test set. The performance of our AI system was compared with each of the nine readers and the average performance of the readers at two modes. **a** B/M (Benign/Malignant) preference mode, **b–d** BI-RADS rating mode. Three BI-RADS ratings were determined by BI-RADS 3 *versus* 4a + , BI-RADS 3,4a *versus* 4b + , and BI-RADS 3,4a,4b *versus* 4c + , respectively. Readers were labelled as senior radiologists if with more than 10 years of clinical experience, while marked as junior radiologists if with no more than 2 years of clinical experience.

We compared the performance of MUP-Net with that of the nine radiologists in two ways. First, we compared the deep learning model with the sensitivity and specificity of individual readers in four modes (i.e., one B/M score and three BI-RADS scores). As shown in Fig. 2, most of the readers were below the ROC curve of the model, indicating a non-inferior performance of our proposed AI system. Second, we compared the performance of MUP-Net with average performance of junior and senior radiologists. The senior radiologists showed their strengths over AI only in the BI-RADS 4a+ and 4b+ scores. By contrast, AI had a superior performance over the average performance of junior radiologists in all four modes, which was in line with our expectation. The greatest advantage of AI over average performance of junior radiologists was in the BI-RADS 4b+ mode. A higher operating point selection resulted in a lower false positive rate on the premise of sacrificing sensitivity as indicated in Fig. 2 (b-d).

### AI-assisted reader study

An AI-assisted reader study was conducted to evaluate the advantage of the domain knowledge-based interpretable AI system in guiding clinical decision-making. To achieve this, in addition to the same paired multimodal US images and the corresponding malignancy risk probability, the matched representative prototypes as well as AI-generated individual contribution scores from each prototype candidate were reviewed by the readers. Figure 2 showed that 26 out of 36 persons (9 persons in each decision mode) were above the ROC curves of the AI, which was an obvious improvement over previous reader studies without any AI assistance. Such a phenomenon was particularly apparent between junior radiologists and senior radiologists. Specifically, senior radiologists obtained an improvement of 7.6% sensitivity in B/M score and 6.9% specificity in BI-RADS 4a+ score. By contrast, junior radiologists gained more benefits, including an 11.3% improvement of sensitivity in BI-RADS 4b+ score, and a 6.3% improvement of specificity in BI-RADS 4a+ score.

Table 2 summarizes the changes in BI-RADS rating and biopsy rate made by the readers in completing the AI-assisted reader study. For benign lesions, most readers (7 out of 9) preferred to avoid at least 3.4% to a maximum of 16.7% unnecessary biopsies along with a better BI-RADS rating (5 out of 9 downgraded more BI-RADS categories). In terms of malignant lesions, all readers (9 out of 9) decided to upgrade more BI-RADS categories as well as most readers (8 out of 9) suggested more biopsies. These results demonstrated great potential of our AI to assist radiologists in making better clinical assessment, especially for junior radiologists.

**Table 2:** Summary of changes in clinical outcomes made by readers R1-R9 in completing the AI-assisted reader study

	Benign lesions (*N* = 60)					Malignant lesions (*N* = 60)				
	Adjustments			Biopsy rate		Adjustments			Biopsy rate	
	Maintain	Upgrade^a^	Downgrade^b^	Solo	+AI	Maintain	Upgrade^a^	Downgrade^b^	Solo	+AI
R1^**^ (13 yrs)	54	4	2	53.3%	56.7%	49	6	5	100%	100%
R2^*^ (2 yrs)	32	8	20	70.0%	53.3%	36	17	7	98.3%	98.3%
R3 (5 yrs)	53	4	3	41.7%	36.7%	31	27	2	90.0%	93.3%
R4 (3 yrs)	35	8	17	88.3%	83.3%	24	26	10	100%	100%
R5^**^ (18 yrs)	52	3	5	36.7%	33.3%	55	4	1	96.7%	95.0%
R6 (3 yrs)	47	1	12	56.7%	43.3%	47	11	2	95.0%	95.0%
R7^**^ (10 yrs)	56	2	2	71.7%	71.7%	41	17	2	100%	100%
R8^*^ (1 yrs)	42	7	11	36.7%	31.7%	25	29	6	71.7%	78.3%
R9^*^ (1 yrs)	54	5	1	40.0%	43.3%	37	20	3	73.3%	86.7%

^a^Shows the number of lesions that the radiologists altered for either an increased BI-RADS or a preference change from “benign to malignancy” under the same BI-RADS. ^b^Shows the number of lesions that the radiologists altered due to either a decreased BI-RADS or a preference change from “malignancy to benign” under the same BI-RADS. Considering BI-RADS 4a+ as a test positive for malignancy, the readers suggest ‘biopsy’ without (Solo) and with (+AI) computer assistance. ^**^and^*^ indicate the reader belongs to a senior radiologist or a junior radiologist, respectively.

Table 3 summarizes the effectiveness of the interpretable AI in aiding radiologists in the matter of accuracy increment. It was observed that none of the readers suffered from a descending accuracy in BI-RADS ratings and B/M preference. As a clinical decision-supporting system, our AI system was more beneficial for junior radiologists rather than senior radiologists (i.e., 5.3% and 7.5% *versus* 2.0% and 2.5% in BI-RADS rating and B/M preference, respectively).

**Table 3:** Summary of the effectiveness of interpretable AI in aiding readers R1-R9

	Changes of accuracy in different decision modes				Questionnaire of AI assistance			
	Mode^a^	Solo	+ AI	Increment	Q1	Q2	Q3	Q4
R1^**^ (13 yrs)	BI-RADS	73.3%, 80.0%, 72.5%	71.7%, 83.3%, 71.0%	+ 0.1%	98.3%	88.3%	80.0%	Y
	B/M	84.2%	84.2%	+ 0.0%				
R2^*^ (2 yrs)	BI-RADS	64.1%, 77.5%, 73.3%	71.7%, 82.5%, 78.3%	+ 5.9%	70.8%	93.3%	62.5%	Y
	B/M	70.8%	77.5%	+ 6.7%				
R3 (5 yrs)	BI-RADS	74.1%, 71.6%, 55.0%	78.3%, 85.8%, 63.3%	+ 8.9%	77.5%	96.7%	74.2%	Y
	B/M	75.0%	80.8%	+ 5.8%				
R4 (3 yrs)	BI-RADS	55.8%, 77.5%, 70.0%	58.3%, 82.5%, 80.0%	+ 5.8%	92.5%	85.0%	70.0%	Y
	B/M	79.1%	82.5%	+ 3.4%				
R5^**^ (18 yrs)	BI-RADS	80.0%, 81.6%, 64.1%	80.8%, 85.0%, 65.0%	+ 1.7%	10.8%	87.5%	80.8%	Y
	B/M	85.0%	85.0%	+ 0.0%				
R6 (3 yrs)	BI-RADS	70.0%, 79.1%, 74.1%	75.8%, 84.2%, 80.0%	+ 5.6%	71.7%	93.3%	63.3%	Y
	B/M	70.0%	75.8%	+ 5.8%				
R7^**^ (10 yrs)	BI-RADS	64.1%, 73.3%, 64.1%	64.2%, 80.0%, 70.0%	+ 4.2%	81.6%	85.8%	75.0%	Y
	B/M	73.3%	80.8%	+ 7.5%				
R8^*^ (1 yrs)	BI-RADS	67.5%, 57.5%, 52.5%	73.3%, 69.2%, 54.2%	+ 6.4%	79.2%	81.7%	59.2%	Y
	B/M	63.3%	75.8%	+ 12.5%				
R9^*^ (1 yrs)	BI-RADS	66.6%, 64.1%, 56.6%	71.6%, 69.2%, 57.5%	+ 3.7%	84.2%	76.7%	81.7%	Y
	B/M	62.5%	65.8%	+ 3.3%				

^a^BI-RADS mode includes 3 *versus* 4a + , 3,4a *versus* 4b+ and 3,4a,4b *versus* 4c+ while B/M mode only contains benign versus malignancy. Considering 4a + , 4b + , 4c+ in BI-RADS mode and ‘M’ in B/M mode as test positive for malignancy, the performance of radiologists in the AI-assisted ready study is calculated without (Solo) and with (+AI) computer assistance, respectively. The increment here represents the average accuracy improvement in both BI-RADS and B/M modes. The detailed descriptions of Q1-Q4 are listed in Fig.1. ** and * indicate the reader belonging to a senior radiologist or a junior radiologist, respectively.

We demonstrated the advantageous use of domain knowledge-based explainable features rather than malignancy risk probability, from two aspects. First, a questionnaire was surveyed to summarize readers’ subjective attitudes towards AI assistance. Second, we performed an additional test by presenting only the AI-predicted probability of malignancy with no explainable features. Supplementary Table 5 compares two types of AI assistance on the clinical test set. The better clinical assessment made by the readers indicates the advantage of providing explainable features over just providing the malignancy probability predicted by the AI.

### The importance of explainable features in clinic

**Fig. 3:**
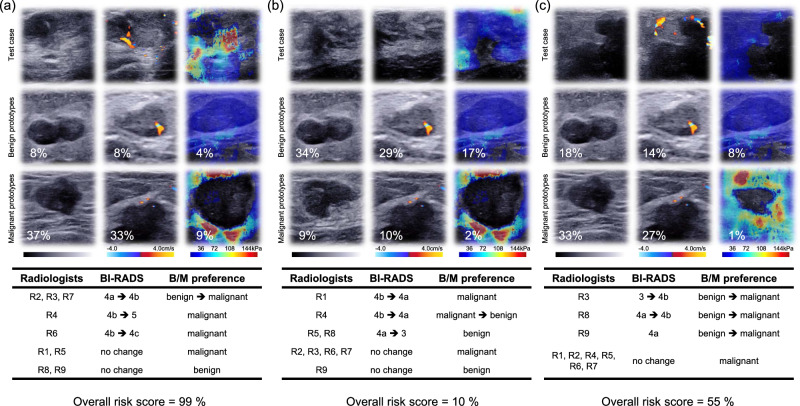
Three test cases to illustrate the adjustment of nine readers with the assistance of AI. For each case, six representative prototypes and corresponding normalized contribution scores were presented to readers. For either benign or malignant set, representative prototypes were selected from prototype candidates (see Supplementary Fig. 6) with the highest contribution scores. **a** and **c** are pathology-confirmed malignant lesions, while **b** is a pathology-confirmed benign lesion. R1-R9 represents the nine readers, respectively.

Compared to previous black-box deep learning models (e.g., heatmaps listed in Supplementary Fig. 5), our proposed MUP-Net learned representative cases (i.e., prototypes listed in Supplementary Fig. 6) from each modality to perform a similarity comparison during inference, resulting in a better way of human-machine interaction. We have listed three examples in Fig. 3 to demonstrate how domain knowledge-based explainable features could benefit radiologists in clinical practice.

Figure 3 (a) is a malignant lesion with a high predicted malignancy risk probability (i.e., the contribution scores of the malignant prototypes were much higher than those of the benign prototypes). Except for R8 and R9, all other radiologists either increased their BI-RADS ratings or altered their B/M preference from benign to malignant, indicating the ability of the AI to optimize radiologists’ preferences. When a lower overall cancer risk probability was presented to a benign lesion as shown in Fig. 3 (b), most radiologists tended to improve their original decisions. Figure 3 (c) is a malignant lesion with an ambiguous risk score − 55%, which was difficult to convince radiologist in clinical practice. However, explainable features (i.e., the highest contribution score 33% belonging to a malignant prototype from US B-mode) still helped 3 out of 9 readers make positive adjustment towards a higher risk of malignancy.

The detailed adjustments made in the AI-assisted reader study was listed in Supplementary Tables 6-14. It should be noted that the inaccurate malignancy risk probability was inevitable in the AI system. To diminish the impact of erroneous prediction, domain knowledge-based prototypes and similarity scores have the potential to provide a second-level validation. In particular, if the AI had an opposite preference to the ground truth result, the majority of the readers would give a negative answer to Q1 and Q3 of our questionnaire. In other words, radiologists preferred to agree with the learned prototypes, instead of recognizing the overall prediction of the AI. Another interesting finding of our study was that the distribution of contribution scores conformed with the clinical experience in utilizing multimodal US images. To be specific, US B-mode took a dominant role in image interpretation, while the US colour Doppler was an important supplement. As a newly proposed modality in BI-RADS guidelines, the contribution score of US elastography was relatively weaker than the other two modalities under the same category of prototypes. These observations implied the potential clinical applicability of our AI system, following the same radiomic analysis routine.

## Discussion

Accurate determination of breast cancer risk from multimodal US images could considerably improve patients’ outcomes and avoid unnecessary biopsies^26^. To accelerate a broader adoption of deep learning technology by human experts in clinical practice, we propose a domain knowledge-based interpretable AI system that not only provides an overall cancer risk prediction, but also offers an efficient human-machine interaction. We demonstrate that our AI system has the potential to assist clinicians, especially for junior radiologists, in making better and confident decisions.

Deep learning frameworks have previously demonstrated its superiority over hand-crafted features in medical imaging fields as a clinical decision-supporting system^38^. However, the black-box nature of deep learning has hindered the establishment of the trust from human experts, even though its performance has reached or surpassed human experts^39,40^. In other words, clinicians could either simply trust the output of AI for its plausibility or overrule it, making the clinical value of AI controversial. To address the lack of interpretability in deep learning, post-hoc explainability approaches have recently been adopted by outputting intermediate results such as saliency maps^41,42^ and heatmaps^29^. However, these methods cannot clearly explain the inner working mechanism of the model while utilizing these cues.

Herein, we propose a domain knowledge-based interpretable AI system on multimodal US images namely MUP-Net, which exploits its inherent explainability by making comparison among representative cases (i.e., prototypes) during inference, and incorporates with clinical domain knowledge of breast US images for prototype selection. The focus of our AI system is to explore an understandable AI decision-making strategy for radiologists as a novel approach of human-machine collaboration. The proposed MUP-Net balances the interpretability of conventional machine learning with the superior performance of deep learning.

The performance of our AI system has been demonstrated in three aspects. First, the interpretable MUP-Net achieved comparable performance with prevalent black-box models. For instance, no significant difference was observed between MUP-Net and those off-the-shelf methods, such as VGG, ResNet, and DenseNet families on the validation cohort. Second, in the reader study, the majority of the readers were below the ROC curve of AI system in terms of sensitivity and specificity. Our MUP-Net showed a superior performance over junior radiologists, while maintaining a competitive performance with senior radiologists. Third, such an interpretable AI system could help avoid unnecessary biopsies for BI-RADS 4 rating patients, which is the major challenge in clinical practice^43,44^. It is important to note that BI-RADS 4 lesions occupied over 85% of our dataset, making it more challenging and representative to clinical need.

In addition to the overall improvement in performance, the AI-assisted reader study revealed the enhancement of clinical applicability brought by the explainable features. On one hand, if the AI system outputted a correct prediction the same as the radiologist’s assessment, it would increase the confidence level of the radiologist. One important evidence is that all readers gave positive feedbacks in Q4, indicating their agreements with the reasoning process of AI. On the other hand, when MUP-Net outputted an inaccurate malignant probability, which did exist in clinical practice, our questionnaire demonstrated that the inherent explainable features have potential to alleviate such a discrepancy. Specifically, a positive answer to Q2 as well as negative answers to Q1 and Q3 revealed the potential of the explainable features to help readers adhere to their original decisions. Another finding of our study is that the advance of AI-assisted improvement in sensitivity and specificity occurred on junior radiologists rather than senior radiologists, suggesting the potential educational applicability of this interpretable AI system in training or assisting junior clinicians.

Moreover, the domain knowledge is applied to supervise the learning process of AI system, which ensures the quality of output explainable features. The key point is to supervise the MUP-Net to select discriminative benign and malignant cases as prototypes for generating credible explainable features presented to readers. The domain knowledge for supervision is based on the standard BI-RADS Atlas and physician observations in clinical practice, which restricts the range of prototype candidates to a subset of BI-RADS. It is interesting to note that the learned contribution scores followed the radiomic analysis routine in clinical practice with a relatively weaker weight of US elastography than the other two modalities under the same category of prototypes.

There are a few limitations in our study. Our dataset is exclusively acquired using Aixplorer US scanners and does not include the variability generated from various scanner manufacturers. Therefore, the proposed AI system may not achieve the same high performance in the external cohorts. In addition, more data are needed for further optimization and testing across diverse clinical settings to demonstrate the usefulness and generalizability of our system. Moreover, our MUP-Net is an image-only deep learning system. To further improve our system, we should include patients’ medical histories and demographics as metadata input. This information should be relevant and beneficial for comprehensive cancer risk prediction.

### Supplementary information

Supplementary Information

Description of Additional Supplementary Files

Supplementary Data 1

Supplementary Data 2

Reporting Summary

## Data Availability

The main data supporting the results of this study are available within the paper and its Supplementary Information. Source data underlying Fig. 2, Tables 2–3 can be found in Supplementary Data 1 and 2. Because of the patient privacy, raw ultrasound datasets from The First Affiliated Hospital of Anhui Medical University and Xuancheng People’s Hospital of China cannot be available for public release. However, data in the reader study can be available for academic study from the lead corresponding author (Xuejun Qian) on reasonable request, subject to permission from the institutional review boards of the hospitals.

## Code availability

The codes used in this study are available at https://github.com/Qian-IMMULab, and are provided in Zenodo with the identifier: 10.5281/zenodo.11046790. The following packages were used: python 3.9.0, PyTorch 1.13.0, Torchvision 0.14.0, Numpy 1.23.4, Scikit-learn 1.1.3, Statsmodels 0.14.0, and Matplotlib 3.6.1. Code for preprocessing the data from raw ultrasound images is available for research purpose upon a request to the lead corresponding author (Xuejun Qian).
