# Supplementary material for: A domain knowledge-based interpretable deep learning system for improving clinical breast ultrasound diagnosis
Source: Commun Med (Lond). 2024 May 17;4:90. doi: 10.1038/s43856-024-00518-7 (PMC11101659; doi:10.1038/s43856-024-00518-7)
Supplement: Supplementary file 1 — Supplementary Information [file 43856_2024_518_MOESM1_ESM.pdf]

A domain knowledge-based interpretable deep learning system for improving clinical breast ultrasound diagnosis

Lin Yan, Zhiying Liang, Hao Zhang, Gaosong Zhang, Weiwei Zheng, Chunguang Han, Dongsheng Yu, Hanqi Zhang, Xinxin Xie, Chang Liu, Wenxin Zhang, Hui Zheng, Jing Pei, Dinggang Shen, and Xuejun Qian

Table of content

Supplementary Fig. 1 | Overview of the prospective workflow. 3

Supplementary Fig. 2 | The schematic architecture of the interpretable deep learning network. 4

Supplementary Fig. 3 | Performance comparison between MUP-Net and other deep learning models. 5

Supplementary Fig. 4 | Performance comparison when different modality features are randomly altered by certain ratios. 6

Supplementary Fig. 5 | Heatmaps generated by Grad-CAM and Grad-CAM++ on three sample cases. 7

Supplementary Fig. 6 | The learned prototypes in MUP-Net for each modality. 8

Supplementary Table 1 | Performance evaluation on the validation cohort. 9

Supplementary Table 2 | Cross validation on the validation cohort. 10

Supplementary Table 3 | Performance evaluation on the test cohort. 11

Supplementary Table 4 | Performance evaluation when different modality features are randomly altered by certain ratios. 12

Supplementary Table 5 | Evaluation of two types of AI assistance on clinical test set. 13

Supplementary Table 6 | Details of the adjustments made by reader R1 (13 years of experience) in completing the AI-assisted reader study. 14

Supplementary Table 7 | Details of the adjustments made by reader R2 (2 years of experience) in completing the AI-assisted reader study. 15

Supplementary Table 8 | Details of the adjustments made by reader R3 (5 years of experience) in completing the AI-assisted reader study. 16

Supplementary Table 9 | Details of the adjustments made by reader R4 (3 years of experience) in completing the AI-assisted reader study. 17

Supplementary Table 10 | Details of the adjustments made by reader R5 (18 years of experience) in completing the AI-assisted reader study. 18

Supplementary Table 11 | Details of the adjustments made by reader R6 (3 years of experience) in completing the AI-assisted reader study. 19

Supplementary Table 12 | Details of the adjustments made by reader R7 (10 years of experience) in completing the AI-assisted reader study. 20

Supplementary Table 13 | Details of the adjustments made by reader R8 (1 year of experience) in completing the AI-assisted reader study. 21

Supplementary Table 14 | Details of the adjustments made by reader R9 (1 year of experience) in completing the AI-assisted reader study. 22

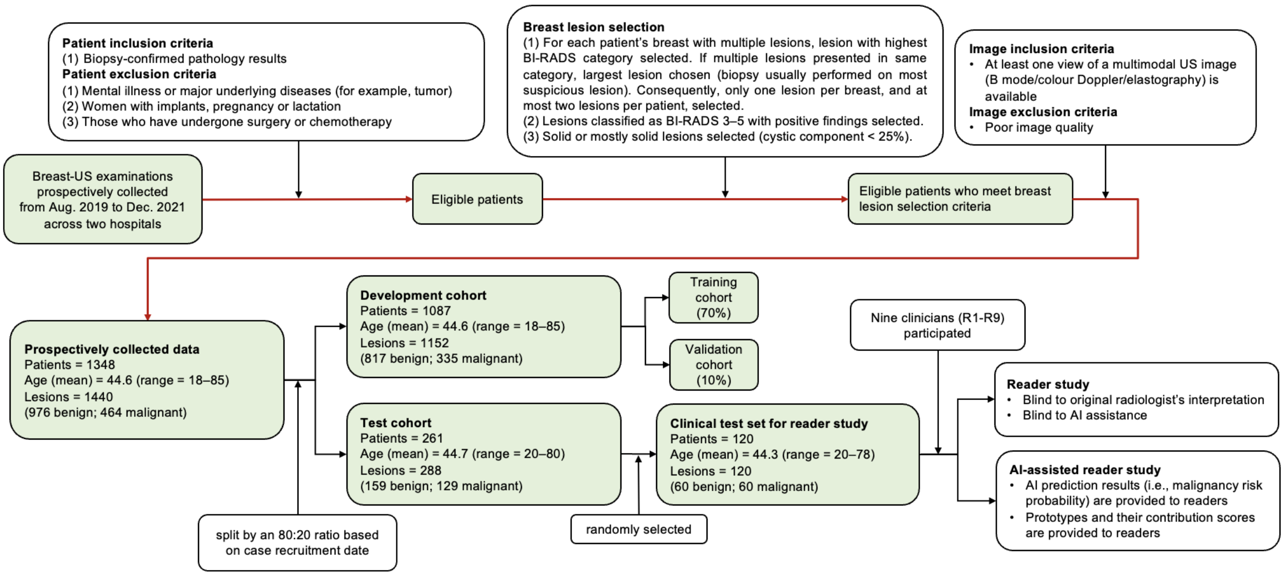

Supplementary Fig. 1 | Overview of the prospective workflow. The AI system was developed from our ultrasound dataset prospectively collected between August 2019 and December 2022. The data was split by an 80%:20% ratio for model development and test based on case recruitment date. For development cohort, the samples were further split into a training cohort (70%) and a validation cohort (10%) for model optimization. For test cohort, we randomly selected 120 lesions from 120 patients to perform the reader study.

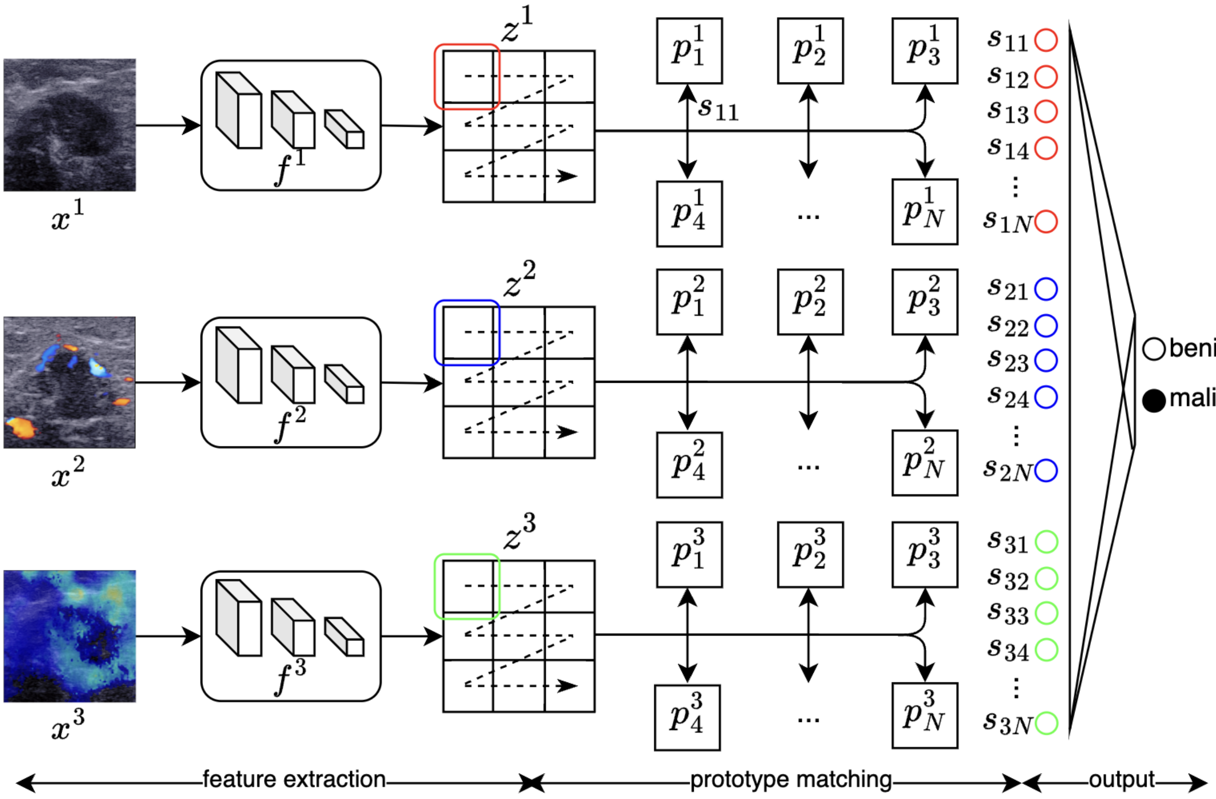

Supplementary Fig. 2 | The schematic architecture of the interpretable deep learning network. Each pathway of the deep learning network extracts informative features through an independent ResNet-18 backbone f^m. The similarity scores between image features and learned prototypes are generated by making a comparison between each distilled image patches and prototypes p_i^m. Scores from three modalities are concatenated and classified by a subsequent fully connected layer to perform decision-making.

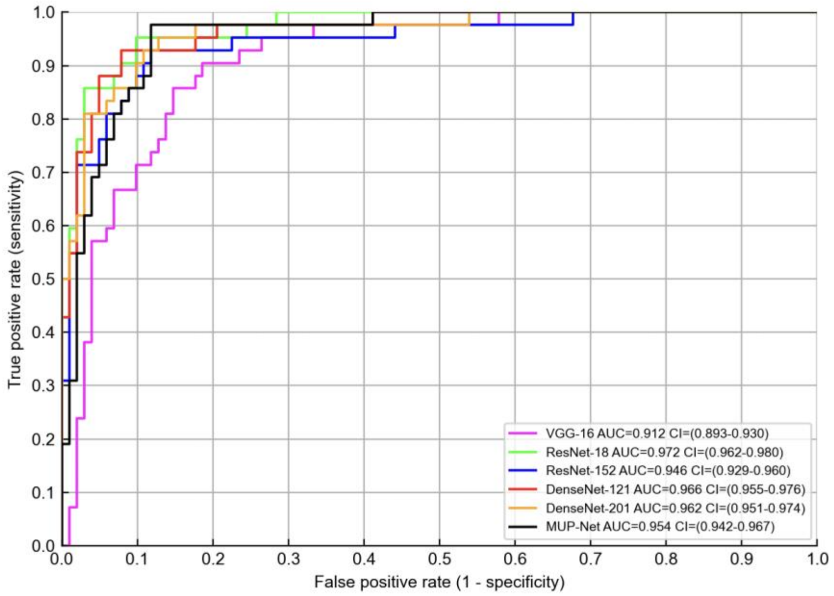

Supplementary Fig. 3 | Performance comparison between MUP-Net and other deep learning models. A total of six deep learning models were compared on the validation cohort, including our MUP-Net (10 prototypes learned for each modality), VGG-16, ResNet-18, ResNet-152, DenseNet-121, and DenseNet-201.

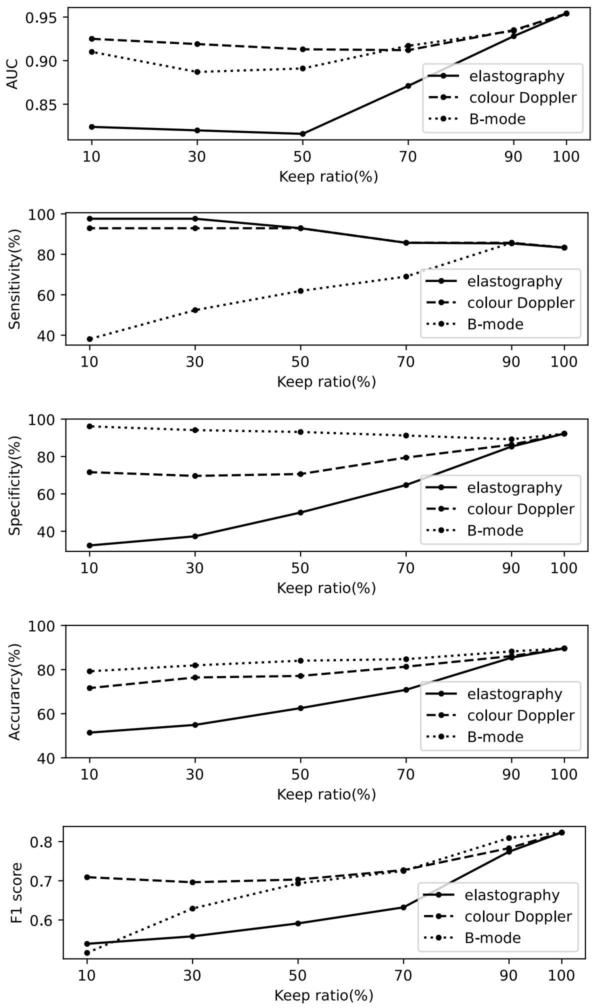

Supplementary Fig. 4 | Performance comparison when different modality features are randomly altered by certain ratios. In the validation cohort, the corresponding modality input of the last layer was filled with zeros. The results were the averaged of 5 times evaluation.

<table>
<tr><th>#</th><th>Modality</th><th>Input image</th><th>Grad-CAM heatmap</th><th>Grad-CAM++ heatmap</th><th>Grad-CAM heatmap on input</th><th>Grad-CAM++ heatmap on input</th></tr>
<tr><td rowspan="3">1</td><td>US B-mode</td><td colspan="5">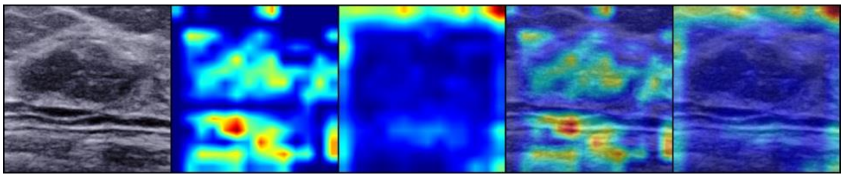</td></tr>
<tr><td>US colour Doppler</td><td colspan="5">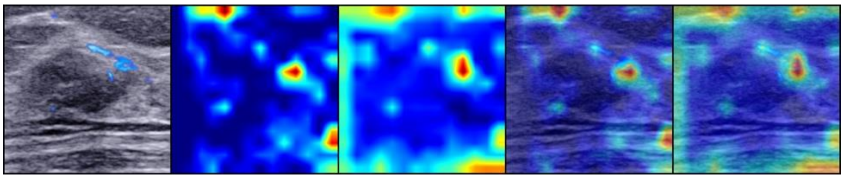</td></tr>
<tr><td>US elastography</td><td colspan="5">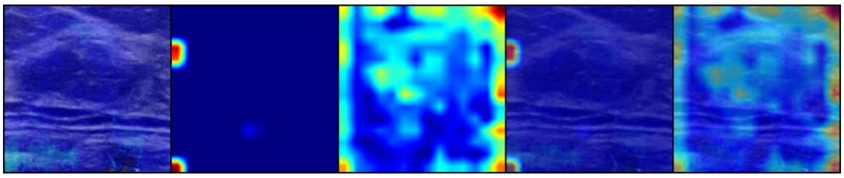</td></tr>
<tr><td rowspan="3">2</td><td>US B-mode</td><td colspan="5">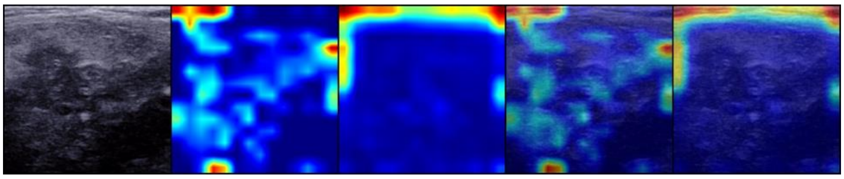</td></tr>
<tr><td>US colour Doppler</td><td colspan="5">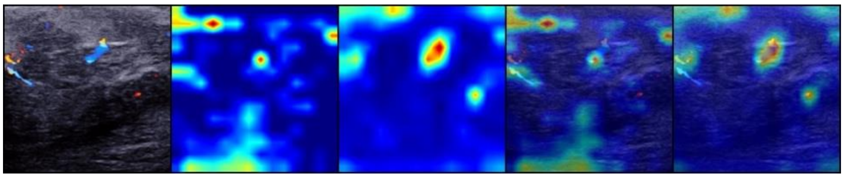</td></tr>
<tr><td>US elastography</td><td colspan="5">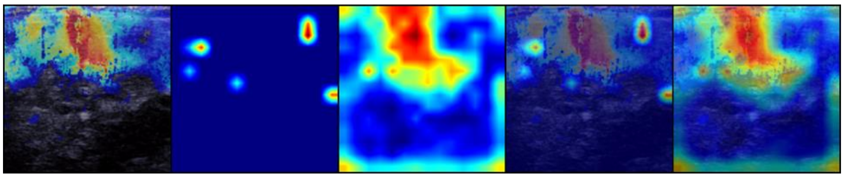</td></tr>
<tr><td rowspan="3">3</td><td>US B-mode</td><td colspan="5">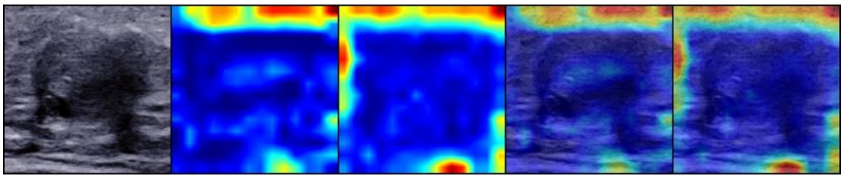</td></tr>
<tr><td>US colour Doppler</td><td colspan="5">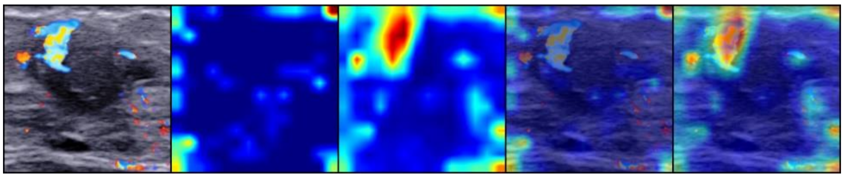</td></tr>
<tr><td>US elastography</td><td colspan="5">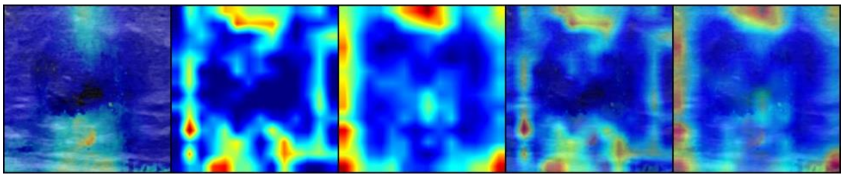</td></tr>
</table>

Supplementary Fig. 5 | Heatmaps generated by Grad-CAM and Grad-CAM++ on three sample cases.

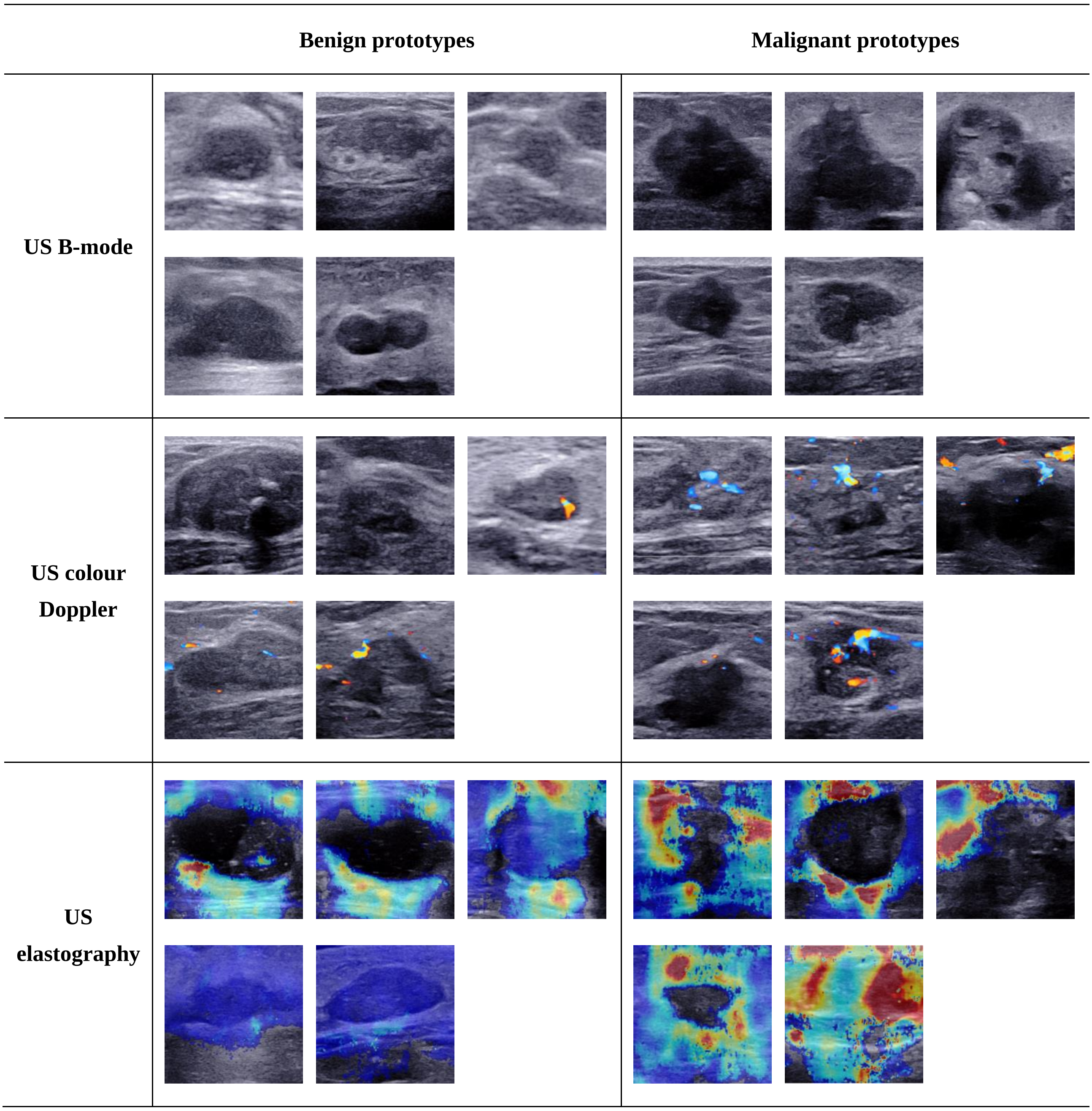

Supplementary Fig. 6 | The learned prototypes in MUP-Net for each modality. Ten prototypes were learned by MUP-Net for each modality. The numbers of benign and malignant cases are fixed and equal in a modality.

Supplementary Table 1 | Performance evaluation on the validation cohort. We evaluated different numbers of prototypes for MUP-Net and some off-the-shelf black box deep learning models on the validation cohort. They were trained on the same training cohort from our dataset.

	**MUP-Net #protos=6**	**MUP-Net #protos=8**	**MUP-Net #protos=10**	**MUP-Net #protos=12**	**MUP-Net #protos=14**
**AUC**	0.916	0.911	0.954	0.930	0.924
**Sensitivity**(%)	80.9	81.0	83.3	78.6	76.2
**Specificity**(%)	87.3	84.3	92.2	91.2	87.3
**Accuracy**(%)	85.4	83.3	89.6	87.5	84.7
**F1 score**	0.764	0.739	0.823	0.785	0.750
	**VGG-16**	**ResNet-18**	**ResNet-152**	**Dense-121**	**Dense-201**
**AUC**	0.912	0.972	0.946	0.966	0.962
**Sensitivity**(%)	81.0	90.5	88.1	90.5	85.7
**Specificity**(%)	85.3	91.2	80.2	92.2	91.2
**Accuracy**(%)	84.0	91.0	89.6	91.7	89.6
**F1 score**	0.747	0.853	0.831	0.864	0.828

Supplementary Table 2 | Cross validation on the validation cohort. We conducted 5-fold cross validation on MUP-Net (#protos=10).

	**1**	**2**	**3**	**4**	**5**	**Average**
**AUC**	0.942	0.930	0.916	0.957	0.950	0.939
**Sensitivity(%)**	77.3	80.8	81.3	83.7	81.9	81.0
**Specificity(%)**	94.0	92.6	89.8	93.9	94.3	92.9
**Accuracy(%)**	88.9	88.5	87.2	90.6	90.3	89.1
**F1 score**	0.809	0.829	0.799	0.851	0.846	0.827

Supplementary Table 3 | Performance evaluation on the test cohort. We evaluated a MUP-Net with 10 prototypes learned for each modality on the test cohort.

	**MUP-Net #protos=10**
**AUC**	0.902
**Sensitivity(%)**	75.2
**Specificity(%)**	91.8
**Accuracy(%)**	84.4
**F1 score**	0.812

Supplementary Table 4 | Performance evaluation when different modality features are randomly altered by certain ratios. On the validation cohort, the corresponding modality input to the last layer was filled with zeros. Results were the averaged of 5 times evaluation.

**Keep ratio of elastography (%)**	**100**	**90**	**70**	**50**	**30**	**10**
**AUC**	0.954	0.928	0.871	0.816	0.820	0.824
**Sensitivity(%)**	83.3	85.4	85.7	92.9	97.6	97.6
**Specificity(%)**	92.2	85.3	64.7	50.0	37.3	32.4
**Accuracy(%)**	89.6	85.4	70.8	62.5	54.9	51.4
**F1 score**	0.823	0.774	0.632	0.591	0.558	0.539

**Keep ratio of colour Doppler (%)**	**100**	**90**	**70**	**50**	**30**	**10**
**AUC**	0.954	0.935	0.912	0.913	0.919	0.925
**Sensitivity(%)**	83.3	85.7	85.7	92.9	92.9	92.9
**Specificity(%)**	92.2	86.3	79.4	70.6	69.6	71.6
**Accuracy(%)**	89.6	86.1	81.3	77.1	76.4	71.6
**F1 score**	0.823	0.783	0.727	0.703	0.696	0.709

**Keep ratio of B-mode (%)**	**100**	**90**	**70**	**50**	**30**	**10**
**AUC**	0.954	0.934	0.917	0.891	0.887	0.910
**Sensitivity(%)**	83.3	85.7	69.0	61.9	52.4	38.1
**Specificity(%)**	92.2	89.2	91.2	93.1	94.1	96.1
**Accuracy(%)**	89.6	88.2	84.7	84.0	81.9	79.2
**F1 score**	0.823	0.809	0.725	0.693	0.629	0.516

Supplementary Table 5 | Evaluation of two types of AI assistance on clinical test set. The comparison was made by reader study on the averaged nine recruited radiologists in two modes (B/M preference mode or BI-RADS rating mode). First, readers were asked to provide BI-RADS rating and B/M preference without computer assistance (Solo). Second, they were exposed to the malignant probability predicted by AI (+AI) without the explainable features. Third, they were exposed to both the malignant probability and the explainable features.

Benigh/Malignant (B/M) preference mode					
	**Solo**	**+AI w/o Explain. Feat.**	**Inc. by Solo (%)**	**+AI**	**Inc. by Solo (%)**
**Accuracy(%)**	73.6	77.7	5.6%	78.6	6.8%
**Sensitivity(%)**	72.8	78.3	7.6%	79.0	8.5%
**Specificity(%)**	74.8	77.0	2.9%	78.4	4.8%
**BI-RADS 4a+ rating mode (BI-RADS 3 *versus* 4a+)**					
	**Solo**	**+AI w/o Explain. Feat.**	**Inc. by Solo (%)**	**+AI**	**Inc. by Solo (%)**
**Accuracy(%)**	68.4	69.2	1.2%	71.8	5.0%
**Sensitivity(%)**	91.9	93.7	2.0%	93.9	2.2%
**Specificity(%)**	44.9	44.8	-0.2%	49.7	10.7%
**BI-RADS 4b+ rating mode (BI-RADS 3,4a *versus* 4b+)**					
	**Solo**	**+AI w/o Explain. Feat.**	**Inc. by Solo (%)**	**+AI**	**Inc. by Solo (%)**
**Accuracy(%)**	73.7	76.9	4.3%	80.0	8.6%
**Sensitivity(%)**	66.9	72.3	8.1%	75.7	13.2%
**Specificity(%)**	80.2	81.3	1.4%	84.2	5.0%
**BI-RADS 4c+ rating mode (BI-RADS 3,4a,4b *versus* 4c+)**					
	**Solo**	**+AI w/o Explain. Feat.**	**Inc. by Solo (%)**	**+AI**	**Inc. by Solo (%)**
**Accuracy(%)**	64.7	67.0	3.6%	68.6	6.0%
**Sensitivity(%)**	35.2	40.0	13.6%	43.3	23.0%
**Specificity(%)**	94.0	93.9	-0.1%	94.1	0.1%

Supplementary Table 6 | Details of the adjustments made by reader R1 (13 years of experience) in completing the AI-assisted reader study.

Patient ID	w/o AI-assisted		AI-assisted		Q1	Q2	Q3
	BI-RADS	B/M	BI-RADS	B/M			
**Benign lesions**							
38	4b	Benign	4a	Benign	yes	yes	yes
85	4b	Malignant	4a	Malignant	yes	no	no
**Malignant lesions**							
12	4a	Benign	4a	Malignant	yes	yes	no
15	4a	Benign	4a	Malignant	yes	yes	yes
18	4a	Malignant	4b	Malignant	yes	yes	yes
33	4a	Benign	4a	Malignant	yes	yes	yes
73	4a	Malignant	4b	Malignant	yes	yes	yes
177	4a	Malignant	4b	Malignant	yes	yes	yes

Supplementary Table 7 | Details of the adjustments made by reader R2 (2 years of experience) in completing the AI-assisted reader study.

Patient ID	w/o AI-assisted		AI-assisted		Q1	Q2	Q3
	BI-RADS	B/M	BI-RADS	B/M			
**Benign lesions**							
9	4a	Benign	3	Benign	yes	yes	yes
11	4a	Benign	3	Benign	yes	yes	no
19	4b	Malignant	4a	Benign	no	yes	no
38	4a	Benign	3	Benign	yes	no	yes
51	4a	Benign	3	Benign	yes	yes	no
53	5	Malignant	3	Benign	yes	yes	yes
57	4b	Malignant	4a	Benign	no	yes	no
58	4a	Malignant	4a	Benign	yes	yes	no
60	4a	Benign	3	Benign	yes	yes	yes
64	4a	Malignant	3	Benign	yes	yes	yes
71	4b	Benign	3	Benign	yes	yes	yes
75	4c	Malignant	4a	Malignant	no	yes	no
77	4a	Benign	3	Benign	yes	yes	yes
80	4b	Malignant	3	Benign	yes	yes	yes
88	4a	Benign	3	Benign	yes	yes	yes
92	5	Malignant	4c	Malignant	yes	yes	yes
93	4b	Malignant	4a	Malignant	yes	yes	no
94	4a	Malignant	4a	Benign	yes	yes	no
104	4b	Malignant	4a	Malignant	no	yes	no
116	4a	Benign	3	Benign	yes	yes	yes
**Malignant lesions**							
1	4b	Malignant	4c	Malignant	yes	yes	no
4	4b	Malignant	4c	Malignant	yes	yes	yes
12	4c	Malignant	5	Malignant	yes	yes	yes
18	4b	Malignant	4c	Malignant	yes	yes	yes
21	4b	Malignant	4c	Malignant	yes	yes	yes
23	4a	Benign	4b	Malignant	yes	yes	yes
26	4b	Malignant	4c	Malignant	yes	yes	yes
30	4a	Malignant	4b	Malignant	no	yes	no
33	4c	Malignant	5	Malignant	yes	yes	yes
72	4c	Malignant	5	Malignant	yes	yes	yes
73	4c	Malignant	5	Malignant	yes	yes	no
83	4b	Malignant	4c	Malignant	yes	yes	yes
98	4c	Malignant	5	Malignant	yes	yes	yes
105	4c	Malignant	5	Malignant	yes	yes	yes
106	4c	Malignant	5	Malignant	yes	yes	yes
110	4b	Malignant	4c	Malignant	yes	yes	yes
112	4b	Benign	4c	Malignant	no	yes	no

Supplementary Table 8 | Details of the adjustments made by reader R3 (5 years of experience) in completing the AI-assisted reader study.

Patient ID	w/o AI-assisted		AI-assisted		Q1	Q2	Q3
	BI-RADS	B/M	BI-RADS	B/M			
**Benign lesions**							
54	4a	Benign	3	Benign	yes	yes	yes
57	4a	Benign	3	Benign	yes	yes	yes
69	4a	Benign	3	Benign	yes	yes	yes
**Malignant lesions**							
12	4a	Malignant	4c	Malignant	yes	yes	yes
13	4a	Benign	4c	Malignant	yes	yes	yes
14	4a	Malignant	4b	Malignant	yes	yes	yes
18	4a	Malignant	4b	Malignant	yes	yes	yes
21	3	Benign	4b	Malignant	yes	yes	yes
22	3	Benign	4b	Malignant	yes	yes	yes
23	4a	Benign	4b	Malignant	yes	yes	yes
26	4a	Malignant	4b	Malignant	yes	yes	yes
33	4a	Benign	4c	Malignant	yes	yes	yes
36	3	Benign	4b	Malignant	yes	yes	yes
44	4a	Malignant	4c	Malignant	yes	yes	yes
59	4a	Malignant	4b	Malignant	no	no	no
66	4a	Benign	4b	Malignant	yes	yes	yes
72	4b	Malignant	4c	Malignant	yes	yes	yes
73	4a	Benign	4b	Malignant	yes	yes	yes
74	4a	Malignant	4b	Malignant	yes	yes	yes
78	4a	Malignant	4b	Malignant	yes	yes	yes
83	4a	Malignant	4b	Malignant	yes	yes	yes
87	4b	Malignant	4c	Malignant	yes	yes	yes
91	4b	Malignant	4c	Malignant	yes	yes	yes
96	4b	Malignant	4c	Malignant	yes	yes	yes
98	4b	Malignant	4c	Malignant	yes	yes	yes
99	4b	Malignant	4c	Malignant	yes	yes	yes
100	4b	Malignant	4c	Malignant	yes	yes	no
101	4b	Malignant	4c	Malignant	yes	yes	yes
113	4b	Malignant	4c	Malignant	yes	yes	yes
117	4a	Malignant	4b	Malignant	yes	yes	yes

Supplementary Table 9 | Details of the adjustments made by reader R4 (3 years of experience) in completing the AI-assisted reader study.

Patient ID	w/o AI-assisted		AI-assisted		Q1	Q2	Q3
	BI-RADS	B/M	BI-RADS	B/M			
**Benign lesions**							
16	4b	Malignant	4a	Benign	yes	no	no
19	4a	Malignant	3	Benign	yes	yes	yes
31	4a	Benign	3	Benign	yes	no	yes
38	4b	Malignant	4a	Benign	yes	yes	yes
41	4a	Benign	3	Benign	yes	yes	yes
49	4a	Benign	3	Benign	yes	yes	yes
54	4b	Malignant	4a	Benign	yes	yes	yes
55	4b	Malignant	4a	Benign	yes	yes	yes
61	4b	Malignant	4a	Benign	yes	yes	no
65	4c	Malignant	4b	Malignant	no	yes	no
67	4b	Malignant	4a	Benign	yes	yes	yes
75	4b	Malignant	4a	Benign	yes	yes	yes
85	4b	Malignant	4a	Benign	yes	no	yes
88	4b	Malignant	4a	Benign	yes	yes	yes
94	4b	Malignant	4a	Benign	yes	yes	yes
104	4b	Malignant	4a	Benign	yes	yes	no
108	4b	Benign	4a	Benign	yes	yes	yes
**Malignant lesions**							
4	4b	Malignant	4c	Malignant	yes	yes	yes
5	4b	Malignant	4c	Malignant	yes	yes	no
6	4b	Malignant	4c	Malignant	yes	yes	yes
7	4c	Malignant	5	Malignant	yes	yes	yes
12	4c	Malignant	5	Malignant	yes	yes	yes
13	4b	Malignant	4c	Malignant	yes	yes	yes
15	4b	Malignant	5	Malignant	yes	yes	yes
18	4a	Malignant	4c	Malignant	yes	yes	yes
21	4c	Malignant	5	Malignant	yes	yes	yes
23	4b	Malignant	5	Malignant	yes	yes	yes
25	4c	Malignant	5	Malignant	yes	yes	yes
26	4b	Malignant	5	Malignant	yes	yes	yes
36	4b	Malignant	4c	Malignant	yes	yes	yes
37	4b	Malignant	4c	Malignant	yes	yes	yes
44	4b	Malignant	4c	Malignant	yes	yes	yes
66	4b	Malignant	4c	Malignant	yes	yes	yes
74	4c	Malignant	5	Malignant	yes	yes	yes
83	4b	Malignant	4c	Malignant	yes	yes	yes
86	4b	Malignant	4c	Malignant	yes	yes	yes
96	4c	Malignant	5	Malignant	yes	yes	yes
100	4b	Malignant	4c	Malignant	yes	yes	no
105	4b	Malignant	4c	Malignant	yes	yes	yes
106	4b	Malignant	4c	Malignant	yes	no	yes
107	4c	Malignant	5	Malignant	yes	yes	yes
113	4b	Malignant	4c	Malignant	yes	yes	yes
117	4b	Malignant	4c	Malignant	yes	yes	yes

Supplementary Table 10 | Details of the adjustments made by reader R5 (18 years of experience) in completing the AI-assisted reader study.

Patient ID	w/o AI-assisted		AI-assisted		Q1	Q2	Q3
	BI-RADS	B/M	BI-RADS	B/M			
**Benign lesions**							
9	4a	Benign	3	Benign	yes	yes	yes
48	4b	Benign	4a	Benign	yes	yes	yes
85	4a	Benign	3	Benign	yes	yes	yes
104	4b	Benign	4a	Benign	yes	yes	yes
108	4b	Benign	4a	Benign	yes	yes	yes
**Malignant lesions**							
12	4a	Malignant	4b	Malignant	yes	yes	yes
18	4b	Benign	4b	Malignant	yes	yes	yes
95	4b	Benign	4b	Malignant	yes	yes	yes
113	4a	Benign	4c	Malignant	yes	yes	yes

Supplementary Table 11 | Details of the adjustments made by reader R6 (3 years of experience) in completing the AI-assisted reader study.

Patient ID	w/o AI-assisted		AI-assisted		Q1	Q2	Q3
	BI-RADS	B/M	BI-RADS	B/M			
**Benign lesions**							
52	4c	Malignant	4b	Malignant	yes	yes	yes
53	4c	Malignant	3	Benign	yes	yes	yes
54	4a	Malignant	3	Benign	yes	yes	yes
57	4b	Malignant	3	Benign	yes	yes	yes
58	4a	Malignant	3	Benign	yes	yes	yes
60	4a	Malignant	3	Benign	yes	yes	yes
64	4a	Malignant	3	Benign	yes	yes	yes
68	4b	Malignant	4a	Malignant	yes	yes	yes
80	4a	Malignant	3	Benign	yes	yes	yes
88	4a	Malignant	3	Benign	yes	yes	yes
93	4b	Malignant	4a	Malignant	yes	yes	no
109	4b	Malignant	4a	Malignant	yes	yes	no
**Malignant lesions**							
4	4b	Malignant	4c	Malignant	yes	yes	no
5	4b	Malignant	4c	Malignant	yes	yes	no
7	4a	Malignant	4b	Malignant	yes	yes	yes
12	4c	Malignant	5	Malignant	yes	yes	yes
18	4b	Malignant	4c	Malignant	yes	yes	yes
21	4b	Malignant	4c	Malignant	yes	yes	yes
23	4b	Malignant	4c	Malignant	yes	yes	yes
26	4c	Malignant	5	Malignant	yes	yes	yes
73	4c	Malignant	5	Malignant	yes	yes	yes
106	4c	Malignant	5	Malignant	yes	yes	yes
107	4c	Malignant	5	Malignant	yes	yes	yes

Supplementary Table 12 | Details of the adjustments made by reader R7 (10 years of experience) in completing the AI-assisted reader study.

Patient ID	w/o AI-assisted		AI-assisted		Q1	Q2	Q3
	BI-RADS	B/M	BI-RADS	B/M			
**Benign lesions**							
65	5	Malignant	4a	Benign	yes	yes	yes
75	4b	Benign	4a	Benign	yes	yes	yes
**Malignant lesions**							
4	4b	Benign	4b	Malignant	yes	yes	yes
10	4a	Benign	4b	Malignant	yes	yes	yes
12	4b	Malignant	4c	Malignant	yes	yes	yes
14	4b	Benign	4c	Malignant	yes	yes	yes
15	4a	Benign	4b	Malignant	yes	yes	no
18	4a	Benign	4b	Malignant	yes	yes	yes
23	4a	Benign	4b	Malignant	yes	yes	no
25	4c	Malignant	5	Malignant	yes	yes	yes
26	4b	Malignant	4c	Malignant	yes	yes	yes
36	4a	Benign	4b	Malignant	yes	yes	yes
37	4b	Benign	4c	Malignant	yes	yes	yes
66	4a	Malignant	4b	Malignant	yes	yes	yes
73	4b	Malignant	4c	Malignant	yes	yes	yes
74	4b	Malignant	4c	Malignant	yes	yes	yes
86	4b	Benign	4b	Malignant	yes	yes	yes
95	4a	Benign	4b	Malignant	yes	yes	yes
105	4b	Benign	4b	Malignant	yes	yes	yes

Supplementary Table 13 | Details of the adjustments made by reader R8 (1 year of experience) in completing the AI-assisted reader study.

Patient ID	w/o AI-assisted		AI-assisted		Q1	Q2	Q3
	BI-RADS	B/M	BI-RADS	B/M			
**Benign lesions**							
19	4a	Malignant	3	Benign	yes	yes	yes
31	4a	Benign	3	Benign	yes	yes	yes
38	4a	Malignant	4a	Benign	yes	yes	yes
54	4a	Malignant	4a	Benign	yes	yes	yes
55	4b	Malignant	4a	Malignant	yes	yes	yes
65	4b	Malignant	4a	Malignant	yes	yes	yes
71	4a	Benign	3	Benign	yes	yes	yes
77	4a	Malignant	3	Benign	yes	yes	yes
79	4a	Benign	3	Benign	yes	yes	yes
85	4a	Benign	3	Benign	yes	yes	yes
88	4a	Malignant	3	Benign	yes	yes	yes
**Malignant lesions**							
0	4a	Benign	4a	Malignant	yes	no	yes
4	4a	Benign	4b	Malignant	no	no	no
5	4a	Benign	4b	Malignant	no	no	no
7	4a	Benign	4a	Malignant	no	yes	no
14	4a	Benign	4a	Malignant	yes	no	no
22	4a	Benign	4b	Malignant	yes	no	no
26	4a	Benign	5	Malignant	yes	yes	yes
30	4a	Benign	4a	Malignant	yes	yes	yes
37	3	Benign	4a	Malignant	no	yes	no
44	4a	Benign	4b	Malignant	no	yes	no
47	4a	Benign	4a	Malignant	yes	yes	no
50	4a	Malignant	4b	Malignant	yes	yes	yes
66	3	Benign	4a	Malignant	no	no	no
72	4b	Malignant	4c	Malignant	yes	yes	no
74	4a	Malignant	4b	Malignant	no	no	no
78	4a	Malignant	4b	Malignant	yes	yes	no
83	3	Benign	4a	Benign	no	no	no
86	3	Benign	4a	Malignant	no	no	no
87	3	Benign	4a	Malignant	no	no	no
90	4a	Benign	4b	Malignant	yes	yes	yes
91	4a	Malignant	4b	Malignant	no	yes	no
95	4a	Malignant	4b	Malignant	no	no	no
96	4a	Malignant	5	Malignant	yes	yes	yes
99	4a	Malignant	4b	Malignant	yes	yes	no
105	4a	Malignant	4b	Malignant	yes	yes	no
106	3	Benign	4a	Malignant	yes	no	no
107	4a	Malignant	4b	Malignant	no	yes	no
113	4a	Malignant	4c	Malignant	yes	yes	no
117	4a	Malignant	4b	Malignant	yes	yes	no

Supplementary Table 14 | Details of the adjustments made by reader R9 (1 year of experience) in completing the AI-assisted reader study.

Patient ID	w/o AI-assisted		AI-assisted		Q1	Q2	Q3
	BI-RADS	B/M	BI-RADS	B/M			
**Benign lesions**							
54	4a	Benign	3	Benign	yes	yes	yes
**Malignant lesions**							
7	4b	Malignant	4c	Malignant	yes	yes	yes
14	3	Benign	4a	Benign	yes	yes	yes
18	3	Benign	4a	Benign	no	no	no
21	4a	Benign	4a	Malignant	yes	yes	yes
22	4a	Benign	4a	Malignant	yes	yes	yes
26	3	Benign	4b	Benign	yes	no	no
37	4a	Benign	4b	Malignant	yes	yes	yes
44	4a	Benign	4b	Malignant	yes	no	yes
47	4a	Benign	4b	Benign	yes	yes	yes
66	3	Benign	4a	Benign	yes	yes	no
73	4a	Benign	4a	Malignant	yes	no	yes
74	3	Benign	4a	Benign	no	no	no
78	4a	Benign	4b	Benign	yes	yes	yes
83	3	Benign	4a	Benign	no	no	no
86	3	Benign	4a	Benign	no	no	no
95	3	Benign	4b	Benign	no	no	no
96	4a	Benign	4b	Malignant	yes	yes	yes
98	4a	Benign	4b	Benign	no	yes	no
99	4b	Benign	4b	Malignant	yes	yes	yes
106	4b	Benign	4b	Malignant	yes	yes	yes
