# Supplementary material for: A domain knowledge-based interpretable deep learning system for improving clinical breast ultrasound diagnosis
Source: Commun Med (Lond). 2024 May 17;4:90. doi: 10.1038/s43856-024-00518-7 (PMC11101659; doi:10.1038/s43856-024-00518-7)
Supplement: Supplementary file 2 — Description of Additional Supplementary Files [file 43856_2024_518_MOESM2_ESM.pdf]

Description of Additional Supplementary Files

File name: Supplementary Data 1

File Description: Fig. 2 data of the main text.

File name: Supplementary Data 2

File Description: Table 2 and 3 data of the main text.
