# Supplementary material for: A domain knowledge-based interpretable deep learning system for improving clinical breast ultrasound diagnosis
Source: Commun Med (Lond). 2024 May 17;4:90. doi: 10.1038/s43856-024-00518-7 (PMC11101659; doi:10.1038/s43856-024-00518-7)
Supplement: Supplementary file 5 — Reporting Summary [file 43856_2024_518_MOESM5_ESM.pdf]

nature portfolio

Corresponding author(s): Xuejun Qian

Last updated by author(s): Aug 17, 2023

Reporting Summary

Nature Portfolio wishes to improve the reproducibility of the work that we publish. This form provides structure for consistency and transparency in reporting. For further information on Nature Portfolio policies, see our Editorial Policies and the Editorial Policy Checklist.

Statistics

For all statistical analyses, confirm that the following items are present in the figure legend, table legend, main text, or Methods section.

n/a	Confirmed	
☐	☒	The exact sample size (*n*) for each experimental group/condition, given as a discrete number and unit of measurement
☐	☒	A statement on whether measurements were taken from distinct samples or whether the same sample was measured repeatedly
☐	☒	The statistical test(s) used AND whether they are one- or two-sided<br>*Only common tests should be described solely by name; describe more complex techniques in the Methods section.*
☒	☐	A description of all covariates tested
☒	☐	A description of any assumptions or corrections, such as tests of normality and adjustment for multiple comparisons
☐	☒	A full description of the statistical parameters including central tendency (e.g. means) or other basic estimates (e.g. regression coefficient) AND variation (e.g. standard deviation) or associated estimates of uncertainty (e.g. confidence intervals)
☐	☒	For null hypothesis testing, the test statistic (e.g. *F*, *t*, *r*) with confidence intervals, effect sizes, degrees of freedom and *P* value noted<br>*Give P values as exact values whenever suitable.*
☒	☐	For Bayesian analysis, information on the choice of priors and Markov chain Monte Carlo settings
☒	☐	For hierarchical and complex designs, identification of the appropriate level for tests and full reporting of outcomes
☐	☒	Estimates of effect sizes (e.g. Cohen's *d*, Pearson's *r*), indicating how they were calculated

Our web collection on statistics for biologists contains articles on many of the points above.

Software and code

Policy information about availability of computer code

Data collection	All patient data were acquired with Aixplorer ultrasound machine (SuperSonic Imagine, Aix-en-Provence, France).<br>A custom annotation tool for labeling the multi-modal ultrasound images, used to label the ROI box which is internally generated by Aixplorer ultrasound system.
Data analysis	Python (version 3.9.0), PyTorch (version 1.13.0), Torchvision 0.14.0, Numpy 1.23.4, Scikit-learn 1.1.3, Statsmodels 0.14.0, and Matplotlib 3.6.1

For manuscripts utilizing custom algorithms or software that are central to the research but not yet described in published literature, software must be made available to editors and reviewers. We strongly encourage code deposition in a community repository (e.g. GitHub). See the Nature Portfolio guidelines for submitting code & software for further information.

Data

Policy information about availability of data

All manuscripts must include a data availability statement. This statement should provide the following information, where applicable:

- Accession codes, unique identifiers, or web links for publicly available datasets
- A description of any restrictions on data availability
- For clinical datasets or third party data, please ensure that the statement adheres to our policy

The main data supporting the results of this study are available within the paper and its Supplementary Information. Because of patient privacy, raw ultrasound datasets from The First Affiliated Hospital of Anhui Medical University and Xuancheng People’s Hospital of China cannot be made available for public release.

However, data in the reader study can be made available for academic study from the lead corresponding author (qianxj@shanghaitech.edu.cn) on reasonable request, subject to permission from the institutional review boards of the hospitals.

Human research participants

Policy information about studies involving human research participants and Sex and Gender in Research.

Reporting on sex and gender	This study is for breast cancer, so all human participants are women.
Population characteristics	Patient data (100% female) were prospectively recruited from August 2019 to December 2022 when doing breast ultrasound examinations in these two hospitals.
Recruitment	With respect to the prospective study, all participants signed an informed consent developed and approved by IRB. We collected breast ultrasound images by using predetermined criteria.
Ethics oversight	The First Affiliated Hospital of Anhui Medical University Ethics Committee and Xuancheng People's Hospital Ethics Committee

Note that full information on the approval of the study protocol must also be provided in the manuscript.

Field-specific reporting

Please select the one below that is the best fit for your research. If you are not sure, read the appropriate sections before making your selection.

☒ Life sciences ☐ Behavioural & social sciences ☐ Ecological, evolutionary & environmental sciences

For a reference copy of the document with all sections, see nature.com/documents/nr-reporting-summary-flat.pdf

Life sciences study design

All studies must disclose on these points even when the disclosure is negative.

Sample size	On the basis of published literature, it is generally agreed that deep learning requires on the order of tens of thousands examples. Thus, we collected as much available data as possible based on the inclusion criteria.
Data exclusions	The exclusion criteria:<br>1. patients have mental illness, major underlying diseases.<br>2. Women with implants.<br>3. done surgery or chemotherapy.<br>4. poor image quality.<br>5. Images with breast lesion were not confirmed by pathology results.
Replication	All our data was prospectively collected from two hospitals under certain protocol.
Randomization	Our dataset was split based on case recruitment date.
Blinding	Radiologists in the clinical evaluation of the models were blinded to the ground truth and were not involved in dataset collect.

Reporting for specific materials, systems and methods

We require information from authors about some types of materials, experimental systems and methods used in many studies. Here, indicate whether each material, system or method listed is relevant to your study. If you are not sure if a list item applies to your research, read the appropriate section before selecting a response.

Materials & experimental systems

n/a	Involved in the study	
☒	☐	Antibodies
☒	☐	Eukaryotic cell lines
☒	☐	Palaeontology and archaeology
☒	☐	Animals and other organisms
☒	☐	Clinical data
☒	☐	Dual use research of concern

Methods

n/a	Involved in the study	
☒	☐	ChIP-seq
☒	☐	Flow cytometry
☒	☐	MRI-based neuroimaging
